# Diversity and Evolution of *pogo* and *Tc1/mariner* Transposons in the Apoidea Genomes

**DOI:** 10.3390/biology10090940

**Published:** 2021-09-20

**Authors:** Yibing Liu, Wencheng Zong, Mohamed Diaby, Zheguang Lin, Saisai Wang, Bo Gao, Ting Ji, Chengyi Song

**Affiliations:** College of Animal Science and Technology, Yangzhou University, Yangzhou 225009, China; yibing-liu@foxmail.com (Y.L.); zongzone@outlook.com (W.Z.); agrical6@yahoo.fr (M.D.); z.lin@yzu.edu.cn (Z.L.); wang850980245@hotmail.com (S.W.); bgao@yzu.edu.cn (B.G.); tji@yzu.edu.cn (T.J.)

**Keywords:** Apoidea, *pogo*, *Tc1/mariner*, transposons, evolution

## Abstract

**Simple Summary:**

In the class of insects, bees are advanced evolutionary groups that play a pivotal role in ecosystem balance, agriculture, and food security. Transposons are the driving factors of genome evolution and speciation, which shape the genomes of almost all organisms. Among them, the *Tc1/mariner* and *pogo* superfamily, as the most widely distributed transposons in nature, are widely reported in insects. However, in bees, the research on transposons has never been systematically reported. Therefore, this study focuses on annotating the transposons of the *Tc1/mariner* and *pogo* superfamily of bees that were sequenced, revealing the distribution, diversity, potential activities, evolutionary patterns, and structural characteristics of transposons in different bees.

**Abstract:**

Bees (Apoidea), the largest and most crucial radiation of pollinators, play a vital role in the ecosystem balance. Transposons are widely distributed in nature and are important drivers of species diversity. However, transposons are rarely reported in important pollinators such as bees. Here, we surveyed 37 bee genomesin Apoidea, annotated the *pogo* and *Tc1/mariner* transposons in the genome of each species, and performed a phylogenetic analysis and determined their overall distribution. The *pogo* and *Tc1/mariner* families showed high diversity and low abundance in the 37 species, and their proportion was significantly higher in solitary bees than in social bees. DD34D/*mariner* was found to be distributed in almost all species and was found in *Apis mellifera*, *Apis mellifera carnica*, *Apis mellifera caucasia,* and *Apis mellifera mellifera,* and *Euglossa dilemma* may still be active. Using horizontal transfer analysis, we found that DD29-30D/*Tigger* may have experienced horizontal transfer (HT) events. The current study displayed the evolution profiles (including diversity, activity, and abundance) of the *pogo* and *Tc1/mariner* transposons across 37 species of Apoidea. Our data revealed their contributions to the genomic variations across these species and facilitated in understanding of the genome evolution of this lineage.

## 1. Introduction

Bees (Hymenoptera: Apoidea), which originated in the early to mid-cretaceous era [1], are the largest and most crucial radiation of pollinators with more than 20,000 described species [2]. Bees play a critical role in ecosystem balance, sustainable agriculture, and food security around the world [3,4]. Most bee species lead a solitary life; only ~10% of bee species are eusocial [3]. In the superfamily of Apoidea, seven extant families are recognized: Megachilidae and Apidae of the long-tongued families Andrenidae, Colletidae, Halictidae, and Melittidae, and Stenotritidae of the short-tongued families [1,5]. Apidae has the largest family size, with >5900 described species, followed by Halictidae and Megachilidae with >4000 species [2]. By contrast, Stenotritidae is the smallest family and includes just 21 recorded species [2]. Melittidae, with 204 known species, is viewed as the most basal bee family [1,6], although some earlier studies have argued that Colletidae is the sister group to the rest of the bees [7,8]. The emergence of different views relates to advances in molecular and genome research, which has substantially changed and continues to change the understanding of classifications and relationships in bees [3,9,10].

Transposons, also called jumping genes, were once considered junk sequences but were later confirmed to play an important role in genome evolution and size [11,12,13,14]. Transposons account for a significant sequence component of eukaryote genomes, in insects ranging from as little as 1% in the *Antarctic midge* [15] to as much as 65% in the *migratory locust* [16]. Transposons can move in the genome and make copies during this movement, and these processes facilitate the ability to invade the genome of almost all organisms and reshape the structure and phenotype of different lineages [17,18,19].

Transposons are usually divided into two types according to the structural organization and mechanism: Class I represents RNA transposons and Class II DNA transposons. DNA transposons have been widely reported and can be divided into three main types: cut-and-paste, peel-and-paste, and self-synthesizing transposons. Widely reported DNA transposons include: *Tc1/mariner*, *pogo*, *hAT*, *PiggyBac*, *CACTA*, *Helitron,* and *PIF-Harbinger*. The *Tc1/mariner* superfamily is a member of the cut-and-paste group that was first discovered in *Drosophila mauritiana* (transposon *mariner*) and *Caenorhabditis elegans* (transposon *C. elegans* number 1, *Tc1*). Generally, *Tc1/mariner* transposon usually has a single open reading frame (ORF) of about 340 amino acids (aa), flanked by two terminal inverted repeats (TIRs) and dinucleotide target site duplications (TSDs) of TA, and it may represent the most widely distributed transposon superfamily in nature [20,21,22]. The arthropods, particularly insects, are more susceptible to the invasion of DNA transposons including diverse *Tc1/mariner* families, and have been suggested as major reservoir hosts for DNA transposons and RNA viruses [21,23,24,25,26,27,28,29,30,31,32,33].

Based on the phylogeny of the DDE-conserved catalytic motif, *Tc1/mariner* is classified into DD34E/*Tc1* [34,35,36,37,38], DD34D/*mariner* [39,40,41,42], DD35E/*TR* [43], DD36E/*IC* [29], DD37D/*maT* [44,45], DD37E/*TRT* [46], DD39D/*GT* [31], and DD41D/*VS* [30]. The *pogo* transposon was first found in flies [47], then more *pogo*-like elements were detected, such as *Tigger* [48] was found in the human genome; *Fot*, *Tan1*, *Pot1*, *Pot2*, *Flipper,* and *Aft1*-transposons were found in the genome of fungi [49,50,51,52,53,54]; *Lemi1* was found in the genome of plants [55]; and *pogo*-like elements were found in the teleost genome [56]. A previous study confirmed that *pogo* and *Tc1/mariner* are two distinct superfamilies [57], but represent a close phylogeny relationship.

The *mariner* element has been reported in studies of the annotation of honeybees and bumblebees [58,59]. However, comparative studies of transposons in the genome of Apoidea species are lacking. Here, we surveyed the genome of 37 bee species in Apoidea; annotated *pogo* and *Tc1/mariner* transposons in the genome of each species; and determined their phylogenetic positions, classification, overall distribution, and structural characteristics. We also investigated the evolutionary patterns of DD29-36D/*Tigger* and DD34D/*mariner* transposons. Our data reveal the evolutionary landscape of *pogo* and *Tc1/mariner* transposons in Apoidea and will add to the understanding of their contributions to the evolution of the Apoidea genomes.

## 2. Materials and Methods

### 2.1. Distribution of pogo and Tc1/mariner within Apoidea

To survey the distribution and diversity of *pogo* and *Tc1/mariner* DNA transposons in 37 sequenced genomes of Apoidea (taxid:34735), we selected several representative transposase sequences from the well-defined families of *pogo* (DD35D/*Passer*, DD35D/*Fot*, DD29-42D/*Lemi*, DD29-59D/*pogoR*, DD36D/*Mover*, DD29-36D/*Tigger*) and *Tc1/mariner* (DD34E/*Tc1*, DD35E/*TR*, DD36E/*IC*, DD37E/*TRT*, DD38E/*IT*, DD34D/*mariner*, DD37D/*maT*, DD39D/*Guest* and DD41D/*VS*) superfamilies and subjected them to a TblastN search against the Apoidea genomes deposited in a whole-genome shotgun database (https://www.ncbi.nlm.nih.gov, accessed on 22 June 2020) with default parameters (1e-10). The information of all genomes in this study is listed in Table S1. Significant hits (80% identity) were extracted with 2000-base pair (bp) flanking sequences, and the transposon boundaries (TIRs) were then determined manually by alignment using the BioEdit program. The copy number of transposon in each genome was estimated by using BLAST (40% coverage and 80% identity) with the obtained transposon sequence. The genomic transposons that contain putatively intact transposase (>300 aa) flanked with TIRs were designated as intact copies. In addition, transposons with very low copy numbers in the genome, which may be false-positive hits resulting from sequence contamination, were verified manually by checking the presence of the host genomic flank sequences of the transposon insertion.

### 2.2. Phylogenetic Analysis and Protein Domain Prediction

For phylogenetic analysis, the conserved DDE domains of the identified transposases were aligned to the representative families of the *pogo* and *Tc1/mariner* transposases separately using MAFFT (v.7.310). The phylogenetic trees were inferred based on the conserved DDE domain using the maximum-likelihood method in the IQ-TREE program. According to the Bayesian information criterion, the best-suited aa substitution model for these data was the LG + G4 model, which was selected by ModelFinder. The reliability of the maximum-likelihood trees was estimated using the ultrafast bootstrap approach with 1000 replicates [29,43]. Information for all representative sequences involved in constructing the phylogenetic tree is listed in Table S2. The phylogenetic tree of host was generated by Timetree (www.timetree.org, accessed on 12 September 2021).

Protein secondary structure predictions were performed using the PSIPRED program (http://bioinf.cs.ucl.ac.uk/psipred, accessed on 1 March 2021). Putative nuclear localization signal (NLS) motifs were predicted using PSORT (https://www.genscript.com/psort.html?src=leftbar, accessed on 1 March 2021). The protein domains were identified using the profile hidden Markov models on the hmmscan online web server (https://www.ebi.ac.uk/Tools/hmmer/search/hmmscan, accessed on 3 March 2021).

### 2.3. Horizontal Transfer and Evolutionary Dynamics Analysis

The pairwise genetic distance between transpsons and the conserved host genes was applied to determine whether these species have experienced HT events. The coding sequences of 32 host genes, including elongation factor1 alpha (*EF1-a*), heat shock cognate 70 (*Hsc70-4*), tubulin beta-3 (*tub3*), and 29 Ribosomal proteins, were used for the comparison with transposon distance to test the HT hypothesis. The three genes (*EF1-a*, *Hsc70-4*, and *tub3*) were used as internal controls by other studies including HT analysis *of transposons* [60,61], while the 29 Ribosomal proteins are considered universally conserved in most species [62], and their accession numbers are listed in Table S3. Species that do not have a complete CDS region of the *EF1-a*, *Hsc70-4*, *tub3*, and 29 universally conserved genes in the US National Center for Biotechnology Information (NCBI) database were not included in these calculations. Multiple alignments of *EF1-a*, *Hsc70-4*, *tub3*, 29 universally conserved genes, and all transposons in Apoidea were created using MAFFT, and the pairwise distances were then calculated using MEGA software (v.7.2.06; pairwise deletion, maximum composite likelihood) based on two aligned files. The species divergence times were estimated using the online Timetree program (http://www.timetree.org, accessed on 12 June 2021). The bar chart was created by Graphpad 8, and then statistical differences were analyzed using a one-factor ANOVA test in SPSS (*25.0*). Significant differences were assessed at *p* < 0.05. The genomic copies of DD34D/*mariner* were annotated by using RepeatMasker (http://www.repeatmasker.org/RMDownload.html, accessed on 20 June 2021) with representative genome of each species, and then calculated the Kimura divergence parameter distance by using the calcDivergenceFromAlign.pl package from RepeatMasker. The Kimura two-parameter distance reflects the transpositional activity of DD34D/*mariner* on a relative time scale per genome [63], and it is generally believed that the younger transposons display lower Kimura divergences [64].

## 3. Results

### 3.1. Diversity and Distribution of pogo and Tc1/mariner Elements in the Apoidea Genomes

**Figure 1:**
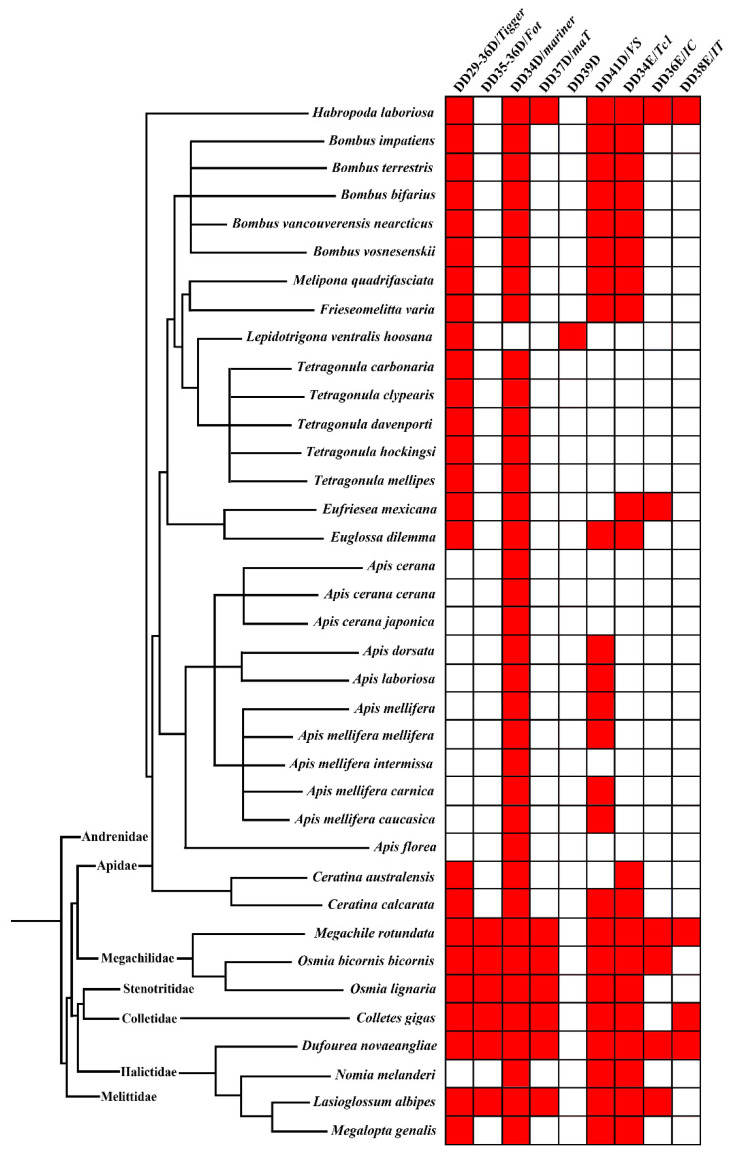
Nine transposon families in various species of Apoidea. The tree on the left is the phylogenetic relationship between all species, generated by Timetree (www.timetree.org, accessed on 2 January 2021), and the block diagram on the right is generated by R studio 1.4.1106, and each red square represents a transposon hit.

To determine the diversity and distribution of *pogo* and *Tc1/mariner* transposons in Apoidea (taxid:34735), we executed a Tblastn search using the NCBI whole-genome shotgun database (https://www.ncbi.nlm.nih.gov, accessed on 22 June 2020) using 15 representative transposase sequences from different families of the *pogo* (DD35D/*Passer*, DD35D/*Fot*, DD29-42D/*Lemi*, DD29-59D/*pogoR*, DD36D/*Mover*, DD29-36D/*Tigger*) and *Tc1/mariner* (DD34E/*Tc1*, DD35E/*TR*, DD36E/*IC*, DD37E/*TRT*, DD38E/*IT*, DD34D/*mariner*, DD37D/*maT*, DD39D/*Guest* and DD41D/*VS*) superfamilies. We identified 164 unique elements in 37 species of Apoidea (Table S4). The phylogenetic tree was then used to define the evolutionary relationships of the *pogo* and *Tc1/mariner* elements in Apoidea that we identified. *TP36_RB* and *Zator* transposases, a clade of the *ITm* group [65], were used as the outgroup, and the incomplete DDE domains were excluded from this analysis. Based on the phylogenetic tree and distribution analysis, two families (DD29-36D/*Tigger* and DD35-36D/*Fot*) were classified as members of the *pogo* superfamily and seven families (DD34E/*Tc1*, DD36E/*IC*, DD38E/*IT*, DD34D/*mariner*, DD37D/*maT*, DD39D/*Guest* and DD41D/*VS*) classified as members of the *Tc1/mariner* superfamily were identified in 37 species of Apoidea (Figure 1 and Figure S4).

**Figure 2:**
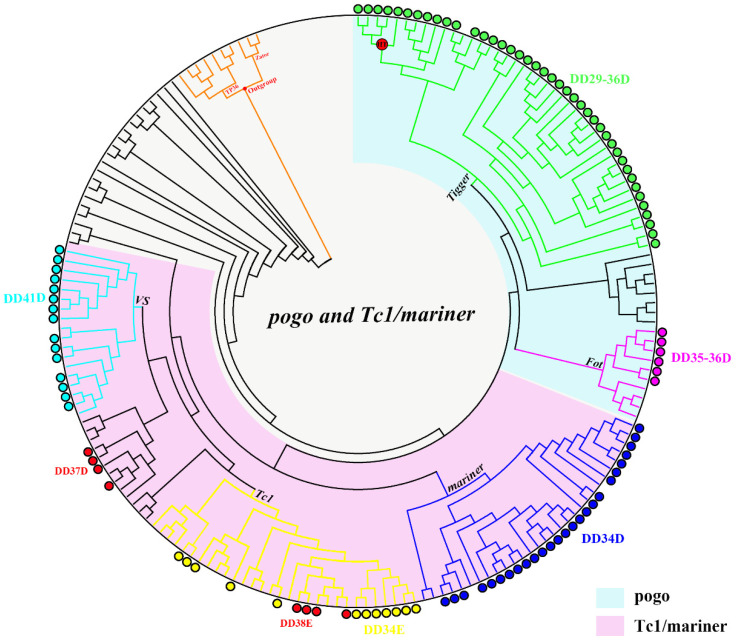
Phylogenetic tree of all transposons. Each color represents a different family, and branches with horizontal transfer (HT) signals are represented by red circles. The transposons found in this study were labeled with circles, and the transposons absent of circle represent the reference sequences of each family.

Differential evolutionary profiles of these families were observed across the Apoidea superfamily in four bee families: Apidae, Megachilidae, Colletidae, and Halictidae (Figure 2 and Table S4). The Apidae family displayed high diversity of species (29 species) but represented a lower diversity of *pogo* and *Tc1/mariner* transposons than the other three families (Megachilidae, Colletidae, and Halictidae) of Apoidea, in which most species contained seven to eight transposon families. Most species of Apidae contained only two to four transposon families, particularly in the *Apis* genus, and most species exhibited only DD34D/*mariner* and DD41D/*VS* families. The exception was *Habropoda laboriosa*, which had one *pogo* family (*Tigger*), but variations of DDE domains (DD30D, DD33E, and DD36D) and six *Tc1/mariner* families (DD34D/*mariner*, DD37D/*MaT*, DD41D/*VS*, DD34E/*Tc1*, DD36E/*IC* and DD38E/*IT*). This species exhibited the highest diversity of *pogo* and *Tc1/mariner* transposons in Apoidea. Four transposon families (DD29-36D/*Tigger*, DD34D/*mariner*, DD41D/*VS,* and DD34E/*Tc1*) were detected in all bumblebees (five species), *Melipona quadrifasciata* and *Frieseomelitta* *varia*, whereas the five species of stingless bee (*Tetragonula davenporti*, *Tetragonula hockingsi*, *Tetragonula carbonaria, Tetragonula clypearis* and *Tetragonula mellipes*) exhibited only the DD29-36D/*Tigger* and DD34D/*mariner* transposon families. The DD29-36D/*Tigger*, DD34D/*mariner*, DD34E/*Tc1,* and DD36E/*IC* families were detected in *Eufriesea mexicana*, and DD29-36D/*Tigger*, DD34D/*mariner*, DD34E/*Tc1,* and DD41D/*VS* families in *Euglossa dilemma*. Moreover, families of DD29-36D/*Tigger*, DD34D/*mariner,* and DD34E/*Tc1* were found in *Ceratina australensis* and *Ceratina calcarata*, and *Ceratina calcarata* also includes a DD41D/*VS* family (Figure 1 and Table S4).

Most species of Megachilidae, Colletidae, and Halictidae displayed high diversity of *pogo* and *Tc1/mariner* transposons, where seven to eight transposon families appeared within each species, except for *Nomia melanderi* and *Megalopta genalis*, where only three and four transposon families were detected, respectively. Eight families of *pogo* and *Tc1/mariner* were detected in *Megachile rotundata* (DD29-36D/*Tigger*, DD35-36D/*Fot*, DD34D/*mariner*, DD34E/*Tc1*, DD36E/*IC*, DD37D/*maT*, DD38E/*IT* and DD41D/*VS*) and *Dufourea novaeangliae* (DD29-36D/*Tigger*, DD35-36D/*Fot*, DD34D/*mariner*, DD34E/*Tc1*, DD36E/*IC*, DD37D/*MaT*, DD38E/*IT* and DD41D/*VS*), which represented the highest diversity across Apoidea species (Figure 1).

### 3.2. Invasions of pogo and Tc1/mariner in the Apoidea Genomes

For further analysis, we investigated the copy numbers of all transposons (Table 1 and Table S4). A copy was defined as Blast result coverage >40% and identity >80%. An intact copy was defined as a Blast result with the full-length of the transposon and the complete ORF encoded. Overall, a total of 164 transposon elements were found in 37 bee species. However, we found that most transposons had low copy numbers in the genome, ranging from 1 to 401 in 37 species, and 115 of 164 transposable elements had <10 copies (>80% identity and >40% coverage) in the host genomes. We found a total of 20 transposons in the *bumble* genus, of which 15 transposons had ≥15 copies and 14 had complete copies. Of the 14 transposons with complete copies, 11 transposons had only one copy (Table 1 and Table S4).

With the exception of DD34D/*mariner*, all other transposon families had relatively low intact copy numbers. For example, neither DD39D/*GT* nor DD36E/*IC* contained an intact copy, whereas, in the DD35-36D/*Fot*, DD37D/*maT,* and DD38E/*IT* families, most transposons contained an intact copy. In addition, *Colletes gigas* had two intact copies in the DD35-36D/*Fot* family; *Colletes gigas* and *Dufourea novaeangliae* had two intact copies in DD37D/*maT*; and *Habropoda laboriosa* had two intact copies in DD38E/*IT*. All intact copies of DD29-36D/*Tigger*, DD41D/*VS,* and DD34E/*Tc1* were ≤5 in hosts. In the DD34D/*mariner* family, we found five species with >10 intact copy numbers: *Apis mellifera*, *Apis mellifera carnica*, *Apis mellifera caucasia*, *Apis mellifera mellifera,* and *Euglossa dilemma.* The intact copy number for the *Euglossa dilemma* was the largest found in this study (Table 1 and Table S4).

**Figure 3:**
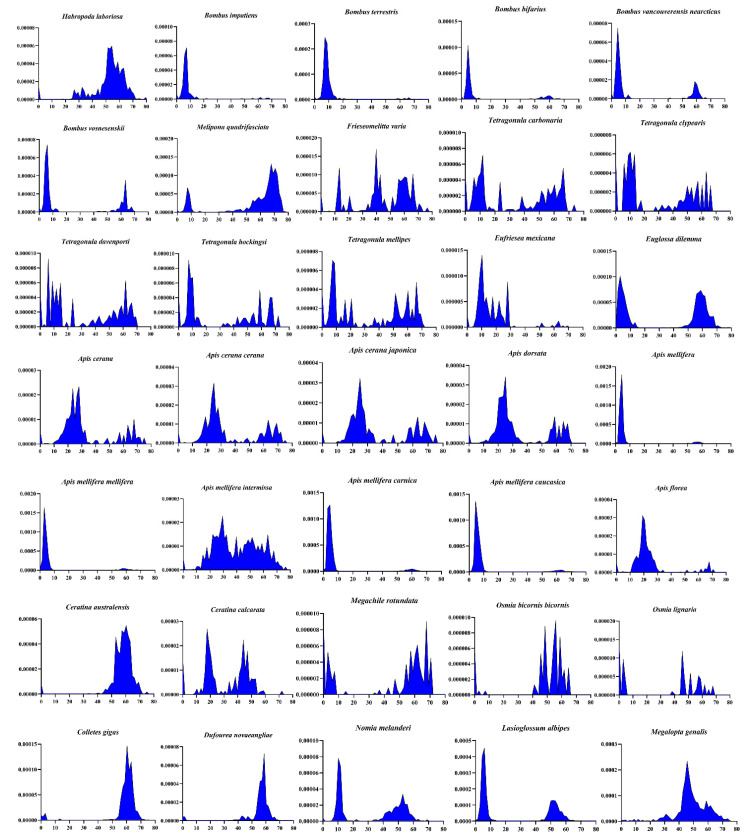
Evolutionary dynamics analysis of DD34D/*mariner* transposons. Species that cannot be executed with RepeatMasker were excluded from this analysis. The *y*-axis represents the percentage of transposon elements in the genome and the *x*-axis indicates the Kimura divergence estimate.

To examine more comprehensively the evolutionary dynamics of transposons in the superfamily, we investigated the DD34D/*mariner* family of transposons, which are distributed in all bees except *Lepidotrigona ventralis hoosana*. Species that cannot be annotated with RepeatMasker were excluded from this analysis. Most transposons of several of the gregarious bee genera, including *Bumble*, *Tetragonula* and *Apis*, seem to have been very young at the time of invasion; some of these species displayed very recent activities (K < 10) (Figure 3), which suggests that these elements are highly active and may still be functional. We also found that the evolutionary dynamics of transposons in each genus are very similar, such as *Bumble* and *Tetragonula*, which means that they have the same accumulation pattern in this genus. However, among *Apis* genera, most European honeybees and Asian honeybees show different patterns of transposon accumulation. In contrast, some European honeybees appear to invade seats earlier than Asian honeybees (Figure 3).

### 3.3. Evolutionary Patterns of pogo and Tc1/mariner in Apoidea

HT is an important form of asexual transmission in nature, and there is increasing evidence that transposons are important participants in HT. Generally, HT events will follow three conditions: (1) high sequence similarity of TEs from divergent taxa; (2) incongruence between TE and host phylogeny; (3) a patchy TE distribution within a group of taxa [66]. In our analysis of four families of Apoidea, namely Apidae, Megachilidae, Colletidae, and Halictidae, the average divergence time was 110 million years ago. We found that *Dufourea novaeangliae*, *Nomia melanderi*, *Lasioglossum albipes,* and *Megalopta genalis* belong to the Halictidae family, but they had obvious irregular distribution of transposons, indicating putative HT events of these transposons across species within this lineage. By contrast, two very close species, *Ceratina calcarata* and *Ceratina australensis*, also had different distribution of transposons. Moreover, some relatively distantly related species on the phylogenetic tree were closer than closely-related species, for example, the DD29-30D/*Tigger* family in *Dufourea novaeangliae* and *Megalopta genalis*, which belong to Halictidae, but *Dufourea novaeangliae* was closer to the *Osmia lignaria* in the Megachilidae family on the clade than to *Megalopta genalis*, which is a more closely-related species of *Nomia melanderi*. Considering these findings, we speculate that some transposons might have been exposed to several episodes of HT.

**Figure 4:**
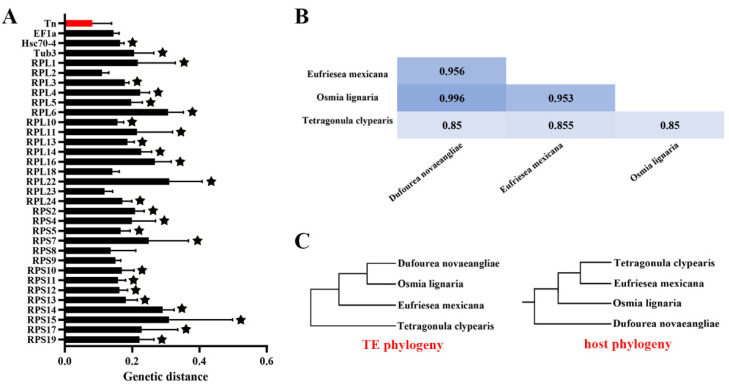
HT analysis of DD29-30D/*Tigger* in Apoidea. (**A**) genetic distance and significance analysis. The *x*-axis represents the paired genetic distance between every two species, and the *y*-axis is the DD29-30D/*Tigger* transposon, *EF1-a*, *Hsc70-4*, *tub3,* and 29 universally conserved ribosomal protein genes. The stars that appear above each bar chart represent a significant difference. (**B**) Sequence identities of DD29-30D/*Tigger* family. The sequence identities were measured by pairwise comparisons of full-length transposases. (**C**) Transposon phylogenetic tree and host phylogenetic tree. The host phylogenetic tree is created by using Timetree (http://timetree.org, accessed on 12 September 2021).

To further illustrate the evolutionary patterns of *pogo* and *Tc1/mariner* in the bee genome, clusters that are inconsistent with the host phylogeny and have an identity of each cluster of the phylogenetic tree close to or greater than 80%, which may experience HT events, are selected. We detected the presence of HT signs in the four clusters (DD29-30D/*Tigger*, DD33D/*Tigger*, DD34D/*mariner*-cluster 1, and DD34D/*mariner*-cluster 2). Then, the pairwise genetic distances between the four host-conserved genes (*EF1-a*, *Hsc70-4*, *tub3* and *RPL3*) and these transposons were calculated and compared. we found that the average distance is smaller for DD29-30D/*Tigger*, DD33D/*Tigger*, DD34D/*mariner*-cluster 1, and DD34D/*mariner*-cluster 2 than for the four host genes (Table S5 and Figure S2). However, our data revealed that with the exception of DD29-30D/*Tigger*, the genetic distances of the other three clusters are close to the host genes, indicating that HT events of these transposons are not well-supported (Table S5). In the cluster of DD29-30D/*Tigger*, we found that the genetic distance is much lower than the host genes and may be exposed to HT events (Table S5). Whereas, the genetic distances varied significantly across host genes and the control gene selection may influence the deduction of HT events. In order to find more evidences to support the HT events of this cluster, we added 29 ribosomal proteins including *RPL3*, which were suggested as universally conserved host genes [62], as controls for further analysis. By calculating the genetic distance of 29 control genes, we found that the average distance was significantly smaller (*p* < 0.05) for DD29-30D/*Tigger* than for almost all host genes, except for *EF1-a*, *RPL2*, *RPL18*, *RPL23*, *RPS8,* and *RPS9* (Figure 4A, Tables S5 and S6). Moreover, the four transposon ORFs of DD29-30D/*Tigger* showed very high overall average sequence identity (91.00% ± 6.00%) across these species (Figure 4B), while their average divergence time was >100 million years ago (Table S5). Additionally, the host phylogeny, by using Timetree and the rebuilt phylogenetic tree of the DD29-30D/*Tigger* cluster, is not consistent (Figure 4C). Overall, these data suggest a possibility of HT of DD29-30D/*Tigger* transposons.

In addition, we did not find HT signals in most other species, which suggests that most of these elements that differ between bee species were not obtained by HT. Overall, these data suggest that the *pogo* and *Tc1*/*mariner* transposons in bees were obtained by both HT and vertical transfer.

### 3.4. Structural Organization of the Detected pogo and Tc1/mariner Transposons

**Figure 5:**
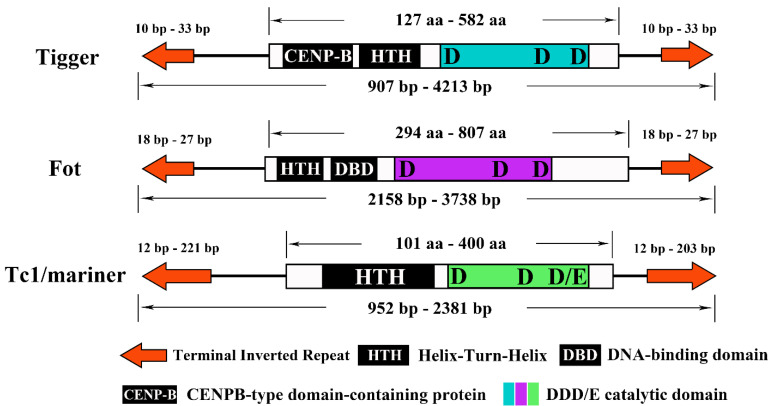
The structure of the two subfamilies of the *pogo* and *Tc1/mariner* transposons. (1) terminal inverted repeats (TIRs) are represented by orange arrows; (2) different protein elements in the DNA-binding domain are represented by black rectangles, each with its name; (3) catalytic domains of the three types of transposons are shown in blue, purple, and green. Amino acid length range, full-length nucleotide length range, and TIR length range are indicated.

Previous studies have shown that the *pogo* and *Tc1/mariner* protein structure is highly conserved along with the transposons, as summarized in Figure 5. The transposase of *Tigger* comprises a CENP-B protein and helix-turn-helix (HTH) domain at the N-terminus and a catalytic domain at the C-terminus. The N-terminal of *Fot-like* transposase comprises HTH and DNA-binding domain regions, and the C-terminal is a catalytic domain. The *Tc1/mariner* transposase contains an HTH domain at the N-terminus and a catalytic domain at the C-terminus. The DD29-36D/*Tigger* transposons invaded almost all species except the *Apis* genus, among which 26 species contain 58 transposons, representing a high diversity of transposons. The length of all transposons ranged from 907 bp to 4213 bp, and the length of the TIR was 10–33 bp. The overall copy number content was very low, but most transposons included an intact copy. Generally speaking, the last aa of the DD/D catalytic domain of the *pogo* transposon was ‘D’, but we found an exception in this study. DD33D and DD33E are similar in structure and have a very close phylogenetic relationship. Considering their phylogenetic location and all species that appear in stingless bees, DD33E may have been caused by the DD33D mutation at some stage. DD34D/*mariner* was found to be the most widely distributed transposon in this study. Almost all bees except *Lepidotrigona ventralis hoosana* have the DD34D/*mariner* transposon and it appears to represent a very young transposon family in Apoidea. We found that all DD34D/*mariner* transposons were around 1200 bp in length and that 24 transposons contained complete transposase. However, among the six *Apis* genomes studied (*Apis cerana*, *Apis cerana cerana*, *Apis cerana japonica*, *Apis dorsata*, *Apis florea,* and *Apis mellifera intermissa*), we could identify only complete ORFs but could not determine the boundary. DD34E/*Tc1* is considered to be the origin of many *Tc1/mariner* transposons and was found to be a very widely distributed transposon in this analysis. All DD34E/*Tc1* transposons were 952–1805 bp in length, and the TIRs were 24–221 bp in length. We also found six *Fot*-like transposons, namely DD35-36D/*Fot* in *Colletes gigas*, *Lasioglossum albipes*, *Megachile rotundata*, *Osmia bicornis bicornis*, *Osmia lignaria,* and *Dufourea novaeangliae*. Six transposons were found in DD36E/*IC*, but they all lost the function of encoding complete transposase. The first five base changes seem to be typical characteristics of some subfamilies. All DD37D/*maT* started with CAGGG, and DD38E/*IT* started with CACTA and CACTG. *Lepidotrigona ventralis hoosana* had an incomplete DD39D/*GT* transposase with a total length of 2381 bp, which retains the typical characteristics of DD39D/*GT* transposon beginning with CTCCC. In addition, the recently well-defined DD41D*/VS* family was also found in this study. Eleven of the 24 transposons had complete ORFs and were 1024–2258 bp in length. All of the bumblebee catalytic domains changed from DD41D to DD40D.

## 4. Discussion

### 4.1. Distribution, Diversity and Copy Number in Apoidea

Transposons can generate new genes through genome rearrangement and overexpression, which play a role in the behavioral diversification of *Apis dorsata* from other honeybees [67]. Given their transposable properties, TEs can accumulate a large number of copies in the host genome, and evidence suggests that transposons are the main cause of differences in genome size between some species [13,14]. The genome of the North African honeybee *Apis mellifera intermissa* contains 5.16% TEs with low diversity, most of which comprise simple repeat sequences [68]. In the European honeybee *Apis mellifera* genome, the percentage of TEs is close to 8% [69]. DNA transposons are the major repeat sequence, and *mariner* transposons are the most common element within this class [70]. A recent study reported that TEs in the genome of the Asian honeybee *Apis cerana* accounted for 9.15% and simple repeats accounted for most TEs [71]. The DNA transposons constitute 0.11% (247 kb) of the *Apis cerana* genome and 0.57% (1.34 Mb) of the *Apis mellifera* genome. The TEs are significantly smaller in *Apis cerana* than in *Apis mellifera* [72]. In a study of bumblebees, TEs accounted for 14.8% and 17.9% in *Bombus terrestris* and *Bombus impatiens*, respectively, and most of them were retrotransposons, of which *Gypsy* was the most common element. The main types of DNA transposons are TIRs of *mariner*, which account for 1.6% and 2.7%, and their abundance is higher than that of *Apis cerana* and *Apis mellifera* [73]. In addition, compared with other Hymenoptera, the abundance of TEs is low in honeybees, and the lack of TEs is one of the main genomic characteristics of these species [74,75,76,77]. Overall, these data suggest that TEs occupy <10% of genomic sequences in honeybees but >10% in bumblebees.

As expected, in our analysis, the transposon diversity was also higher for bumblebees than honeybees. We detected at least seven families of *Tc1/mariner* and two families of *pogo*, which suggests that the genome of some bees represents high diversity of *Tc1/mariner* and *pogo* transposons. In our study, the genomes of 37 bee genomes were rich in *pogo* and *Tc1/mariner* transposons, and included nine different transposon families: DD29-36D/*Tigger*, DD35-36D/*Fot,* DD34D/*mariner*, DD34E/*Tc1*, DD36E/*IC*, DD37D/*maT*, DD38E/*IT*, DD39D/*GT,* and DD41D/*VS*. Among all transposon families discovered to date, DD34D/*mariner* is the most widely distributed and appears in almost all bee genomes. In addition, DD34D/*mariner* is also widely distributed in nature and has been reported in all species [39,78,79,80,81,82]. The distribution of transposons differs between species, but the distribution of transposons is similar in sister species, which suggests that transposons invade the host’s genome before species divergence.

It is generally believed that the copy number is related to transposon activity and that a genome with more intact transposon copies, which include the full-length transposase (no frameshift mutation) and the flanking TIRs with very high identity, usually reflects a recent invasion activity, suggesting that some copies may be still active [83]. We examined the copy numbers for all transposons included in this study and found that most families of transposons had only one or few copies, which suggests that most *Tc1/mariner* and *pogo* transposons do not display significant amplification in these lineages. This is consistent with previous findings that most *Tc1/mariner* is detected with low genomic coverage [68,71]. In addition, we found >10 intact copies of DD34D/*mariner* in the genomes of *Apis mellifera*, *Apis mellifera carnica*, *Apis mellifera caucasia*, *Apis mellifera mellifera,* and *Euglossa dilemma*. The evolutionary dynamics analysis showed that the DD34D/*mariner* elements of these five genomes are very young, and we speculate that these elements may remain active in the genomes of some species. The European honeybee is an important economic animal introduced into China in the last century, where there is a considerable amount of breeding. Active transposon elements may play an important role in the genome evolution and subspecies differentiation of European honeybees, and their existence raises the possibility of molecular operations on honeybees. In addition, the number of newly discovered families of the *pogo* and *Tc1/mariner* superfamily continues to increase as more genomic sequencing data are obtained, and this trend indicates that the diversity of the *pogo* and *Tc1/mariner* superfamilies may be much larger than the currently known families.

### 4.2. Differential Susceptibility of Apoidea Species to DNA Transposons

In this analysis, we detected a total of nine families of *Tc1/mariner* and *pogo* transposons in 37 species, for a total of 164 transposons. Interestingly, we found that the genomes of some solitary bees were rich in DNA transposons. For example, *Habropoda laboriosa* exhibited DD29-36D/*Tigger*, DD34D/*mariner*, DD37D/*MaT*, DD41D/*VS*, DD34E/*Tc1*, DD36E/*IC* and DD38E/*IT* transposons; *Dufourea novaeangliae* exhibited DD29-36D/*Tigger*, DD34D/*mariner*, DD35-36D/*Fot*, DD34E/*Tc1*, DD36E/*IC*, DD37D/*MaT*, DD38E/*IT* and DD41D/*VS* transposons; and *Colletes gigas* exhibited DD29-36D/*Tigger*, DD35-36D/*Fot*, DD34D/*mariner*, DD34E/*Tc1*, DD36E/*IC*, DD37D/*maT*, DD38E/*IT,* and DD41D/*VS* transposons. Social bees exhibited significantly fewer transposons than solitary bees. For example, the *Tetragonula* genus exhibited only two transposon subfamilies, DD29-36D/*Tigger* and DD34D/*mariner*. In *Apis* genus, there are also two families of transposons (DD34D/*mariner* and DD41D/*VS*) that appear in the genome of honeybees. This seems to be related to the living environment. Solitary bees act alone and their long-term migration and living environment are more complicated and unstable, whereas social bees live in a group involving social activities and their living environment is more stable [84]. On further analysis, the *Bombus* genus exhibited four families, DD29-36D/*Tigger*, DD34D/*mariner*, DD41D/*VS,* and DD34E/*Tc1*. Interestingly, *Bombus* genus are a group that once transformed from solitary bees to social bees, which seems to further verify that the content of transposon may be related to the living environment.

We also found another interesting phenomenon. *Apis mellifera*, *Apis mellifera carnica*, *Apis mellifera caucasia,* and *Apis mellifera mellifera* exhibited a complete DD34D/*mariner* element (full-length and complete ORF). However, only the complete ORF of DD34D/*mariner* was exhibited by *Apis cerana*, *Apis cerana cerana*, *Apis cerana japonica*, *Apis dorsata*, *Apis florea,* and *Apis mellifera intermissa*, and the boundary could not be determined. Compared with its close relative, the European honeybee, the Asian honeybee seems to have a stronger selection of transposons in the genome. It is possible that the drones that develop from unfertilized eggs and carry haploid chromosomes experience strong selective pressure on some active elements [85]. In our further analysis of DD41D/*VS*, we only found that *Apis mellifera*, *Apis mellifera carnica*, *Apis mellifera caucasia*, *Apis mellifera mellifera,* and *Apis dorsata* have the complete full-length transposon sequence, but no complete ORF was found. Moreover, *Apis cerana*, *Apis cerana cerana,* and *Apis cerana japonica* did not exhibit any DD41D/*VS* transposon elements. It seems that DD41D/*VS* may be an ancient invasion and that, during long-term genome evolution, European honeybees gradually lost their complete copy and Asian honeybees completely lost the entire transposon. Finally, some studies have noted that a host contains an endogenous enzyme system that can silence the transposon or some virus expression, such as RNA interference [86]. However, the mechanism responsible for the interaction between the disappearance of transposon copies and the host in the honeybee genome requires further research.

### 4.3. HT Events in Apoidea

Transposons are parasitic DNAs whose only role is to replicate and propagate. When a transposon invades a host, the germline genome must be colonized to ensure that it remains in the population. This then increases the copy number [87], and the transposon remains in the genome until, through vertical inhibition, all copies of the transposon become inactive and remain as fossils only [88]. Through genetic drift, these inactive elements can even vanish [88]. To evade this cycle, a transposon must invade a new species or spread to multiple species. That is, to guarantee its longevity, the transposon must transfer to a new genome through HT to start its life cycle again. Although the molecular mechanism responsible for HT is uncertain, studies have provided evidence that HT is the main reason why transposons are widely distributed in nature. Instances include the exchange across marine crustaceans [89], among insects of various orders [90,91], and even between species of different phylae as diverse as the human and parasitic nematode [92].

In this study, although most transposons did not show obvious HT signs, the potential HT traces were detected for DD29-30D/*Tigger*. To find the HT origin of the DD29-30D/*Tigger* transposon, we performed Blast searching of this transposase in *Dufourea novaeangliae* in the NCBI nucleotide collection (nr/nt) database. The complete DDD spacing (DD30D) catalytic domains were detected in *Nosema bombycis*, *Strongyloides stercoralis,* and *Ctenocephalides felis*. Interestingly, these three creatures have parasitic abilities: *Nosema bombycis* is a parasite of silkworm, *Strongyloides stercoralis* is parasitic in humans, and the host of *Ctenocephalides felis* is the cat. These species may serve as potential vectors for *Tigger* transposons that have invaded the bee genome at a certain time; if so, this strongly suggests the existence of a host–parasitic relationship in bees. These findings suggest that the host–parasitic mode may also be the primary mode of HT of *Tigger* transposon.

## 5. Conclusions

Our research provides, for the first time, information about the distribution of *pogo* and *Tc1/mariner* family transposons in 37 sequenced Apoidea genomes. In general, *pogo* and *Tc1/mariner* show high diversity in most species, except for a few honeybees, but have a low abundance. In addition, only DD34D/*mariner* has a high copy number in several species, appears as a complete structure, and may have potential activity. Finally, our results also show that DD29-30D/*Tigger* may experience HT events, and we speculate that they may have invaded their common ancestor before some species formed.

## Figures and Tables

**Table 1:** Copy number information for each transposon family.

	DD29–36D *Tigger*	DD35–36D *Fot*	DD34D *mariner*	DD37D *maT*	DD39D *GT*	DD41D *VS*	DD34E *Tc1*	DD36E *IC*	DD38E *IT*
Copy number	1–67	1–5	1–320	1–6	3	1–401	1–61	3–24	3–50
Intact copy number	1–4	1–2	1–28	1–2	0	1–3	1–5	0	1–2

## Data Availability

The data presented in this study are available on request from the corresponding author.

## Supplementary Materials

The following are available online at https://www.mdpi.com/article/10.3390/biology10090940/s1, Figure S1: phylogenetic tree of TEs based on the alignment of the DDE domains, Figure S2: HT analysis in Apoidea, Table S1: Genome assembly information for all species, Table S2: The access number of reference *pogo* and *Tc1/mariner* elements, Table S3: *EF1-a Hsc70-4 tub3* and Ribosomal protein Accession Number, Table S4: Taxonomic distribution of Apoidea, Table S5: distance of TEs, *EF1-a*, *Hsc70-4*, *tub3*, 29 Ribosomal proteins and Divergence time, Table S6: Significance analysis data.
