# Supplementary material for: Diversity and Evolution of pogo and Tc1/mariner Transposons in the Apoidea Genomes
Source: Biology (Basel). 2021 Sep 20;10(9):940. doi: 10.3390/biology10090940 (PMC8472432; doi:10.3390/biology10090940)
Supplement: Supplementary file 1 [file biology-10-00940-s001.zip › Figure S1-S2.pdf]

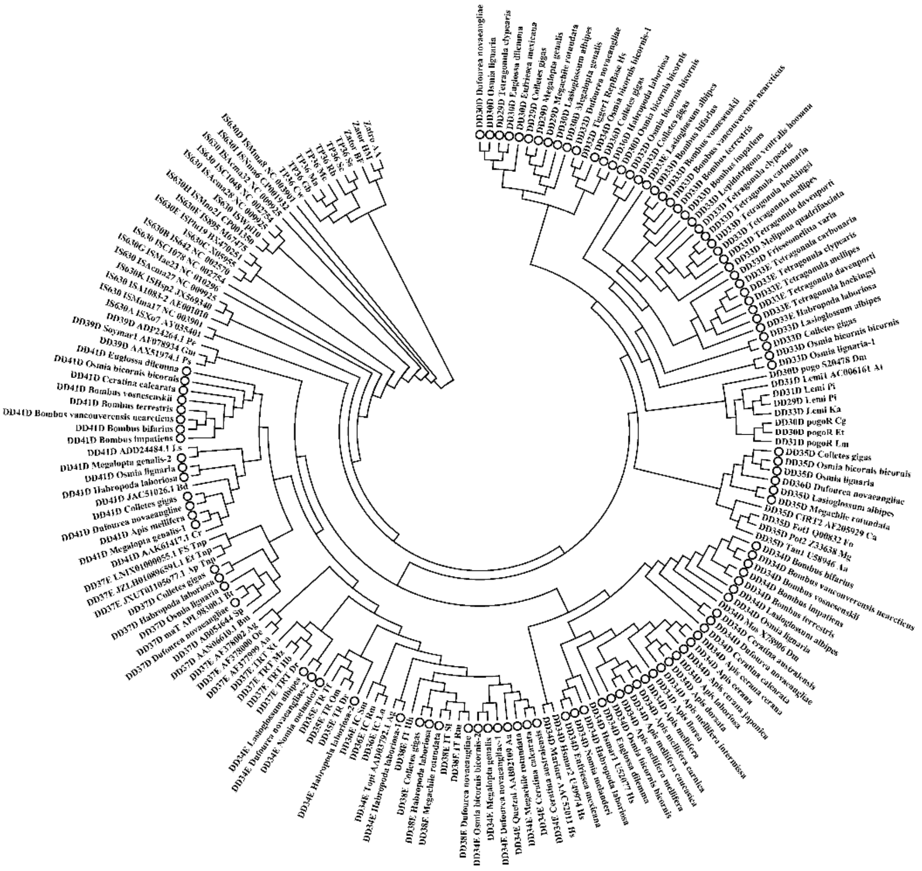

Figure S1. phylogenetic tree of TEs based on the alignment of the DDE domains. Species with only highly fragmented copies and incomplete DD34E motifs in their genome were not included in this analysis. The phylogenetic tree was inferred using the maximum likelihood method with the IQ-Tree program.

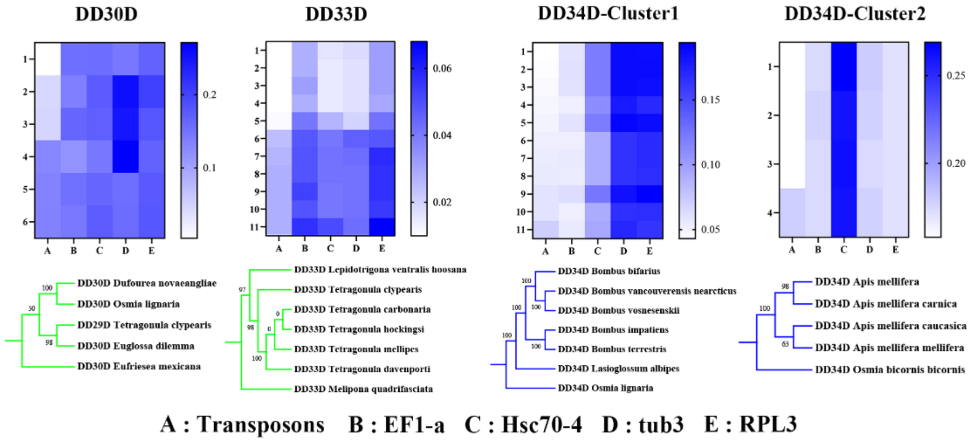

Figure S2. HT analysis in Apoidea. A lighter colour represents a smaller genetic distance, and a darker colour represents a greater genetic distance. The y-axis represents the paired genetic distance between each two species, and the x-axis is the transposon (A) and three different host genes (B: *EF1-a*, C:*Hsc70-4*, D: *tub3* and E: *RPL3*).
